# Blockade of Uttroside B-Induced Autophagic Pro-Survival Signals Augments Its Chemotherapeutic Efficacy Against Hepatocellular Carcinoma

**DOI:** 10.3389/fonc.2022.812598

**Published:** 2022-02-08

**Authors:** Lekshmi R. Nath, Mundanattu Swetha, Vinod Vijayakurup, Arun Kumar Thangarasu, Nair Hariprasad Haritha, Anwar Shabna, Sreekumar U. Aiswarya, Tennyson P. Rayginia, C. K. Keerthana, Kalishwaralal Kalimuthu, Sankar Sundaram, Ravi Shankar Lankalapalli, Sreekumar Pillai, Rheal Towner, Noah Isakov, Ruby John Anto

**Affiliations:** ^1^ Division of Cancer Research, Rajiv Gandhi Centre for Biotechnology, Thiruvananthapuram, India; ^2^ Chemical Sciences and Technology Division, Council for Scientific and Industrial Research (CSIR)-National Institute for Interdisciplinary Science and Technology, Thiruvananthapuram, India; ^3^ Academy of Scientific & Innovative Research (AcSIR), Ghaziabad, India; ^4^ Department of Biotechnology, University of Calicut, Malappuram, India; ^5^ Department of Pathology, Government Medical College, Kottayam, India; ^6^ Department of Surgical Oncology, Jubilee Mission Medical College and Research Institute, Thrissur, India; ^7^ Department of Pathology and Pharmaceutical Sciences, University of Oklahoma Health Sciences Center, Oklahoma, United States; ^8^ The Shraga Segal Department of Microbiology, Immunology, and Genetics, Ben-Gurion University of the Negev, Beer Sheva, Israel

**Keywords:** autophagy, chloroquine, apoptosis, uttroside B, hepatocellular carcinoma, chemoprevention

## Abstract

Our previous study has demonstrated that Uttroside B (Utt-B), a saponin isolated from the leaves of *Solanum nigrum* Linn induces apoptosis in hepatic cancer cells and exhibits a remarkable growth inhibition of Hepatocellular Carcinoma (HCC). Our innovation has been granted a patent from the US (US 2019/0160088A1), Canada (3,026,426.), Japan (JP2019520425) and South Korea (KR1020190008323) and the technology have been transferred commercially to Q Biomed, a leading US-based Biotech company. Recently, the compound received approval as ‘Orphan Drug’ against HCC from US FDA, which reveals the clinical relevance of evaluating its antitumor efficacy against HCC. In the present study, we report that Utt-B promotes pro-survival autophagy in hepatic cancer cells as evidenced by the increased expression of autophagy-related proteins, including LC3-II, Beclin1, ATG 5, and ATG 7, as well as a rise in the autophagic flux. Hence, we investigated whether Utt-B-induced autophagic response is complementing or contradicting its apoptotic program in HCC. Inhibition of autophagy using the pharmacological inhibitors, Bafilomycin A1(Baf A1), and 3-methyl adenine (3-MA), and the biological inhibitor, Beclin1 siRNA, significantly enhances the apoptosis of hepatic cancer cells and hence the cytotoxicity induced by Utt-B. We also found increased expression of autophagy markers in Utt-B-treated xenografts derived from HCC. We further analyzed whether the antimalarial drug, Chloroquine (Cqn), a well-known autophagy inhibitor, can enhance the anticancer effect of Utt-B against HCC. We found that inhibition of autophagy using Cqn significantly enhances the antitumor efficacy of Utt-B *in vitro* and *in vivo*, in NOD SCID mice bearing HCC xenografts. Taken together, our results suggest that the antitumor effect of Utt-B against HCC can be further enhanced by blocking autophagy. Furthermore, Utt-B in combination with Cqn, a clinically approved drug, if repurposed and used in a combinatorial regimen with Utt-B, can further improve the therapeutic efficacy of Utt-B against HCC.

## Introduction

Hepatocellular carcinoma (HCC) is a malignant tumor with limited therapeutic options, about 700,000 people being diagnosed each year (1, 2). Sorafenib, a multitargeted tyrosine kinase inhibitor, which provides only a marginal survival advantage for patients is the major chemotherapeutic option approved by the FDA, against HCC (3–5). We are the first to demonstrate that Utt-B, a saponin isolated in our laboratory from *Solanum nigrum*, induces apoptosis in HCC cells and is ten times more cytotoxic to these cells than sorafenib. The compound inhibits the growth of HCC xenografts developed in NOD/SCID mice and does not induce any pharmacological toxicity (6). This invention has been granted a patent from several countries and the technology has been transferred commercially (https://qbiomed.com/index.php/pipeline/uttroside). Recently, Utt-B received ‘Orphan Drug’ designation against HCC from US FDA (http://prn.to/39oORUp). Since Utt-B exhibits promising antitumor potential against HCC, a comprehensive understanding of its pro-death mechanism is essential to develop new therapeutic approaches against HCC.

Recent reports have demonstrated that autophagy, induced in response to chemotherapeutic agents, has a crucial influence on the clinical outcome of HCC patients (7–9). Autophagy is a catabolic mechanism by which cellular material is delivered to lysosomes for degradation, helping cancer cells to maintain homeostasis by recycling the worn-out cellular components (10). Autophagy, activated in response to chemotherapeutic stress, could manifest as a pro-death mechanism by digesting the cellular contents, consequently aiding in cellular disassembly during cell death. On the contrary, it could act as a crucial factor for delaying cell death, by removing apoptosis-inducing factors such as damaged mitochondria, from the cytosol (11, 12). The role of autophagy, induced in response to chemotherapeutic agents has been reported either as a pro-survival strategy or as a pro-death mechanism in HCC, depending upon the nature of chemotherapeutic insults (13). Therefore, modulating autophagy is an attractive strategy for enhancing the chemotherapeutic potential of antitumor agents against HCC.

In the current study, we demonstrate that, apart from apoptosis, Utt-B induces autophagy also in HCC cells and inhibition of autophagy enhances the anti-HCC activity of Utt-B, revealing the pro-survival role of Utt-B-induced autophagy. These results suggest blockade of autophagy as an attractive strategy for enhancing the chemotherapeutic potential of Utt-B against HCC.

## Materials and Methods

### Collection and Authentication of Plant Materials

*Solanum nigrum* Linn., plants were collected from local areas of Thiruvananthapuram, Kerala and were identified by Dr. G. Valsaladevi, Curator, Department of Botany, University of Kerala, and a voucher specimen has been deposited in the Division of Cancer Research, Rajiv Gandhi Centre for Biotechnology (RGCB) [VOUCHER NO: crp04&crp05]. Fresh plants required for the study were grown in RGCB Garden and collected and shade dried on a monthly basis.

### Isolation and Purification of Utt-B

Isolation and purification of Utt-B is done as previously reported (6).

### Cell Lines

The liver cancer cell line HepG2 was purchased from ATCC (CRL-11997) and Hep3B from NCCS, Pune. Mycoplasma tests were performed on parent cell lines and stable cell lines every 6 months.

### Chemicals and Molecular Biologicals

Important cell culture reagents such as Dulbecco’s Modified Eagle Medium (DMEM) (GIBCO,12800-017) and streptomycin sulfate (GIBCO, 11860-038) were obtained from Invitrogen Corporation (Grand Island, USA). Poly Excel HRP/DAB detection system universal kit (PathnSitu Biotechnologies Pvt. Ltd, India, OSH001) was used for immunohistochemistry experiments. MTT reagent purchased from TCI Chemicals (India) Pvt. Ltd (D0801) and Amersham ECL Plus™ Western blotting reagents (PRPN 2132) were purchased from GE Healthcare Life Sciences (Piscataway, USA). 3-Methyl adenine (M9281), Chloroquine (C6628), Bafilomycin A1 (B1793), DAPI (D9542), Propidium Iodide (P 4170), RNase A (10109142001), Antibodies against Vinculin (V9131), and LC3 (L8918) were procured from Sigma-Aldrich (St. Louis, USA). Antibodies against, β- actin (12620S), LC3 (4599P), p-AMPK α (2531S), AMPK α (2532S), p-mTOR (S2448) (5536S) p-mTOR (S2481) (2974S), mTOR (2972S), Atg5 (8540P), Atg7 (2613P), Beclin-1(3495P), phospho-p70S6Kinase (9205S), p70S6Kinase (2708S), p-4E-BP-1(2855S), 4E-BP-1(9452S), GAPDH (8884S), Akt (9272S) and Beclin siRNA (6222S), Caspase 9 (9508S), Caspase 8 (4790S), PARP (9532S), Cleaved PARP (5625S) were obtained from Cell Signaling Technologies (Beverly, MA, USA) and the antibody against PARP (sc4470), PCNA (sc25280), p-Akt (sc7985-R), Ki67 (sc23900) and Annexin V apoptosis detection kit (sc4252AK) were purchased from Santa Cruz Biotechnology (Santa Cruz, CA, USA). DeadEnd™ Colorimetric TUNEL System from Promega (G7132), PtfLC3 plasmid (21074) was procured from Addgene (Cambridge, MA, USA). All other chemicals were purchased from Sigma Chemicals (St. Louis, MO, USA) unless otherwise mentioned.

### MTT Assay

HepG2 cells were seeded in 96-well plates (2000 cells/well). After overnight incubation, cells were treated with different concentrations of Utt-B and Cqn for 72 h, and cytotoxicity was measured. Fresh media containing 25 μL of MTT solution (5 mg/mL in PBS) was added to the wells and incubated for 2h. At the end of incubation, lysis buffer (20% sodium dodecyl sulfate in 50% dimethyl formamide) was added to the wells (0.1 mL/well) and incubated for another 1h at 37°C. At the end of incubation, the optical density was measured at 570 nm using an ELISA plate reader (Bio-Rad). The relative cell viability in percentage was calculated as (A_570_ of treated samples/A_570_ of untreated samples) X 100. The IC_50_ values were extrapolated from polynomial regression analysis of experimental data.

### Clonogenic Assay

500-1000 cells/well were seeded in 6 well plates and were treated with different concentrations of the compounds (Utt-B &Cqn) for 72h. Then the media was aspirated and fresh media was added and incubated for 1 week. The colonies developed were fixed with glutaraldehyde and stained using crystal violet. The colonies were then viewed under the microscope, photographed and the colonies were counted and the graph was plotted.

### Wound-Healing Assay

A monolayer of confluent HepG2 cells were scratched using a sterile tip to create a wound. Cell debris was gently removed by washing the cells with 1X PBS. Wells were treated with different concentrations of Utt-B and Cqn. Images were taken at different time intervals under a phase contrast microscope.

### Acridine Orange Staining for Acidic Vesicular Organelles (AVOs)

HepG2 cells (2x10^3^ cells) were seeded in 96 well plates and treated with the indicated concentration of Utt-B and incubated for 24h. The cells were rinsed with 1x PBS twice. The treated cells were then stained with acridine orange, which was added at a final concentration of 1µg/mL and incubated for 15 min, washed with PBS, and immediately photographed using a fluorescent microscope (14).

### Beclin siRNA Transfection

HepG2 was transiently transfected with Beclin siRNA and control siRNA using Lipofectamine LTX Plus reagent kit according to manufacturer’s protocol (Invitrogen, USA). 0.35 x 10^6^cells per well were seeded in a six-well tissue culture plate containing 2ml antibiotic-free normal growth medium supplemented with FBS and the cells were incubated to attain 60% confluency and transfected using the Lipofectamine LTX and Plus Reagent Kit (Invitrogen, USA) according to manufacturer’s protocol. The silencing of Beclin expression was confirmed by Western blotting with anti-beclin-1 and the transfection efficiency was standardized at 50-60h before the drug treatment and Utt-B was treated for 8-12h.

### Confocal Microscopy

The pGFP-mRFP-LC3B (ptf-LC3) vector was purchased from Addgene (21074). Briefly, the plasmid present in the bacterial pellet was isolated according to the manufacturer’s instruction (GenElute™ Plasmid Miniprep Kit- Sigma-Aldrich). HepG2 cells were transfected transiently with tandem repeats of GFP-RFP tagged LC3 (ptfLC3) using the Lipofectamine LTX and Plus Reagent Kit (Invitrogen, USA) according to the manufacturer’s protocol. HepG2 cells were seeded at a density of 0.25x 10^6^ in a coverslip and were treated with Utt-B for 24h after transfection, examined, and photographed. DAPI staining was performed by 10 min incubation with 1µg/ml DAPI in HepG2 cells treated with mentioned concentrations of Utt-B and Cqn and viewed under the confocal microscope.

### Detection of Apoptosis by Annexin V Fluorescence Microscopy

Apoptotic cells were detected with the help of a fluorescent microscope by Annexin V FITC kit (sc4252AK) using manufacturer’s protocol (Santa Cruz, CA, USA). Briefly, the cells were seeded in 96-well plates and treated with Utt-B and Cqn as in MTT assay, but for 16h. The cells were then washed with PBS, followed by 1X assay buffer, after which, 0.5-5 μL (0.1-1 μg) of Annexin V FITC per 100 μL assay buffer was added. After incubating for 15 min at room temperature in the dark, the cells were washed with PBS and immediately photographed using a fluorescent microscope, Nikon inverted fluorescent microscope (TE-Eclipse 300).

### Estimation of Apoptosis by FACS

The extent of apoptosis induced by Utt- B and Cqn was estimated by FACS using the Annexin V apoptosis kit (sc4252AK, Santa Cruz, CA, USA). HepG2 cells were seeded in 60 mm culture plates, incubated with different concentrations of drugs. After 16h, cells were trypsinized and pelleted down by low-speed centrifugation, washed with PBS, and were suspended in 1X assay buffer. To the buffer, 5 µL of FITC conjugated Annexin V and 10 µL of propidium iodide were added and incubated for 15 min in dark at room temperature. The cells were then analyzed immediately by flow cytometry to get the percentage of apoptotic cells (FACS Aria™, BD Bioscience).

### Cell Cycle Analysis

HepG2 cells were seeded in 60 mm culture dishes and incubated overnight following which they were treated with different concentrations of Utt-B and Cqn and incubated for 48 h. after incubation, cells were trypsinized, pelleted, and fixed using 70% ethanol. The cells were then treated with 5 µl (10mg/ml) RNase A and incubated for 30 min at 37°C after which 10 µl propidium iodide (10mg/ml) was added and the contents were filtered and analyzed using FACS Aria™ flow cytometer (BD Bioscience).

### Western Blotting Analysis

Cells were treated with the indicated concentration of Utt-B and Cqn for different time points. The cell lysates were then taken and electrophoresed by SDS/PAGE. After this, the proteins from SDS/PAGE were electrotransferred to a membrane, which was then blocked with 5% dried milk for 60 min. The membrane was then washed three times for 5 min each with TBST wash buffer and immunoblotted with the appropriate antibodies overnight at 4^0^C. The membrane was then incubated with HRP-conjugated secondary antibodies for 60 min. The bands were visualized using an enhanced chemiluminescence kit following the manufacturer’s protocol.

### Animal Experiments

#### *In Vivo* Xenograft Model

Tumor xenograft experiments were conducted according to the protocol IAEC/538/RUBY/2016, under the approval from Institutional Animal Ethics Committee, Rajiv Gandhi Centre for Biotechnology. Six-week-old NOD-SCID (NOD.CB17-Prkdcscid/J) male mice is used for the study. Tumors are generated by subcutaneous injection into the right lower flank with 5×10^6^ HepG2 in matrigel. Two weeks after cell injection, the mice will be separated into 4 groups of 6 each. Group, I was treated with vehicle alone, Group II received an intraperitoneal injection of Utt-B in PBS at 5 mg/kg body weight on alternate days, Group III received an intraperitoneal injection of Cqn in PBS at a dose of 60 mg/kg body weight on alternate weekly and Group IV received both Cqn and Utt-B. Drug treatment was continued up to 1 month, animals were euthanized and the tissue samples were collected for further analyses. Tumor dimensions will be recorded three times per week with a digital caliper starting with the first day of treatment (15, 16).

### Group Drug Regimen

- IP injection of the vehicle alone (on alternate days).
- IP injection of Utt-B 5 mg/kg body weight dissolved in PBS (on alternate days).
- IP injection of Cqn 60 mg/kg body weight dissolved in PBS (on alternate days).
- IP injection of Cqn 60 mg/kg & Utt-B 5 mg/kg body weight (on alternate days).

### Toxicological Analyses

The toxicological analysis of the Cqn alone and the combination was performed in 6-8 weeks old female Swiss albino mice as per protocol (IAEC/537/RUBY/2016) approved by the Institutional Animal Ethics Committee, Rajiv Gandhi Centre for Biotechnology.

*Acute toxicity*: The test group will be administered with Cqn (0, 30, 60 &120, mg/kg body weight) and a combination of Utt-B and chloroquine (10/30,10/60, 5/60, and 5/120 mg/kg body weight) as a single dose to groups of six mice each. Animals were euthanized on day 15. The liver tissue was analyzed by histopathology using H&E staining and the serum was used to perform Liver Function Test and Renal Function Test.

*Sub-chronic Toxicity*: Doses of Cqn (0, 30, 60 &120 mg/kg body weight) and a combination of Utt-B and Cqn (10/30, 10/60, 5/60, and 5/120 mg/kg body weight) were given to groups of six mice each. Animals were euthanized after 90 days and toxicity was measured as described above.

### Histology and Immunohistochemistry

The tumor and liver tissues from mice were fixed and sectioned and stained using Hematoxylin and eosin. Immunolocalization of specific proteins in the tissue sections was done using Poly Excel HRP/DAB detection system universal kit for mouse and rabbit primary antibodies (OSH001, PathnSitu Biotechnologies Pvt. Ltd, India) as per manufacturer’s protocol. All the immunohistochemistry images were taken in DMi8 Inverted Fluorescence Research Microscope with DMC 2900 Digital Camera.

### TUNEL Assay

TUNEL assay was performed to detect apoptosis in formalin fixed, paraffin-embedded xenograft tumor tissue sections using Dead End Colorimetric TUNEL System (G7132, Promega) following the manufacturer’s instructions.

### Statistical Analysis

For the flow cytometry, data analysis was performed using the BD FACS Diva software version 5.0.2. H-scoring for Immunohistochemistry and the quantification of western blot were carried out using ImageJ software. The statistical analysis was performed using Graph Pad Prism software (Graph Pad Software Inc., San Diego, CA, USA). Statistical significance was defined as *p <* 0.05. The error bars represent ± SD, taken from the three independent experiments.

## Results

### Uttroside B Induces Autophagy in Hepatic Cancer Cells

**Figure 1:**
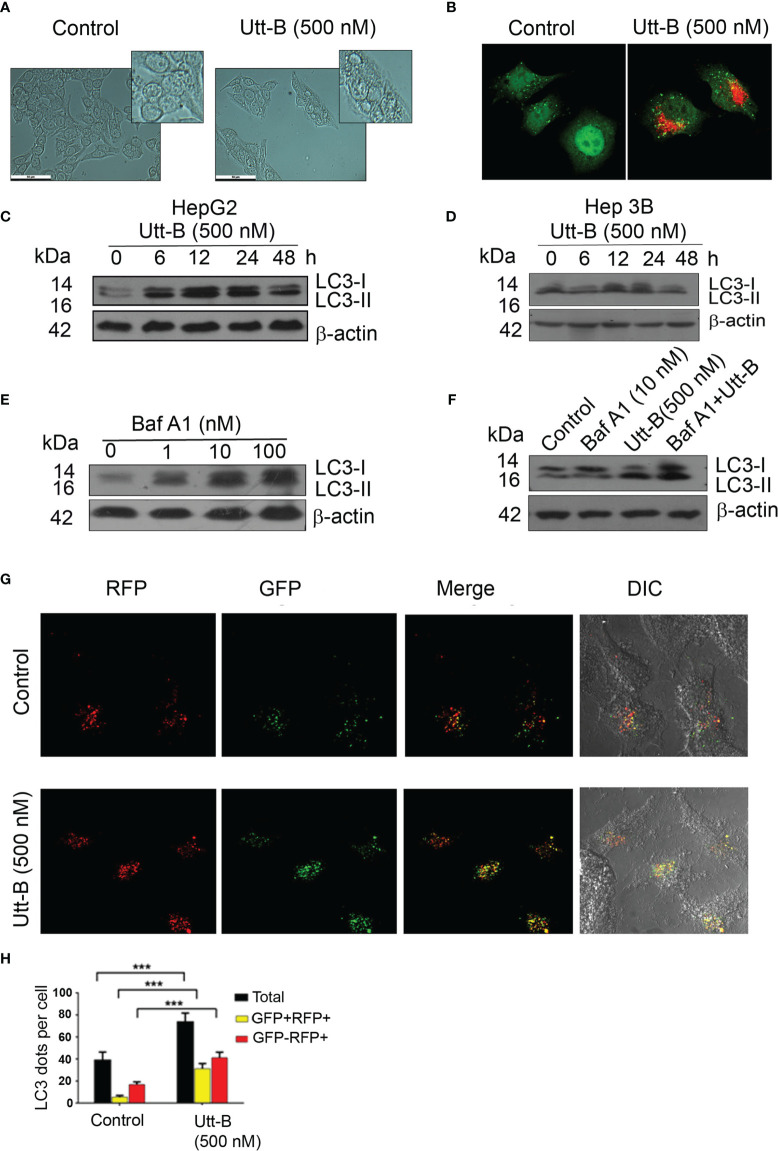
Utt-B induces autophagy*, in vitro*. **(A)** Utt-B treatment induces the formation of vacuolated structures in HepG2 cells. **(B)** Acidic vacuoles are stained with Acridine Orange **(C)** Western blot results indicate that LC3II expression is induced in HepG2 cells from 6h to 12h in HepG2 cells. **(D)** Western blot results indicate that LC3II expression is induced in HepG2 cells from 12h to 24h in Hep3B cells. **(E)** 10 nM Baf A1 is enough to block autophagy in HepG2 cells, as inferred from LC3-II accumulation. **(F)** Co-treatment of Utt-B and Baf A1 increases LC3-II accumulation. **(G, H)** Accumulation of LC3-II by Utt-B was quantitated by RFP-GFP-LC3 tagged protein assay. *** level of significance 3.

Our previous study had demonstrated that Utt-B, a saponin isolated from the leaves of *Solanum nigrum* Linn exhibits exceptional antitumor efficacy against hepatic cancer cells (6). We noticed that the IC50 concentration of Utt-B induces vacuolated structures in HepG2 cells (**Figure 1A**) and when stained with acridine orange, exhibit a bright red fluorescence, indicating an increase in the formation of acidic vesicles (**Figure 1B**). Both were preliminary evidence for the induction of autophagy, a controlled self-digestive process capable of influencing the death of tumor cells. To confirm Utt-B -induced autophagy in liver cancer cells, the expression pattern of the autophagosome marker, LC3II, was studied in two hepatic cancer cell lines, HepG2 and Hep3B having different HBV statuses. While LC3II expression is induced in HepG2 cells from 12 h onwards and remains elevated up to 24 h (**Figure 1C**) on Utt- B treatment, the increased expression starts from 6 h and remains elevated up to 48 h, in Hep3B cells (**Figure 1D**). An increase in autophagosome formation is a strong indication of autophagy. However, blockage of autophagosome processing in any step leading from autophagosome maturation to its fusion with lysosomes (i.e., Autophagy blockage) could also lead to autophagosome accumulation and increased LC3II formation. Hence, to see whether Utt-B -induced LC3II over-expression is due to induction of autophagy or blockage of autophagosome processing, we co-treated Utt-B with Baf A1, a tool for correctly deciphering the reason for LCII increase (17, 18). Baf A1 itself causes the accumulation of LC3II by blocking the fusion of autophagosome and lysosome. If Utt-B is an inducer of autophagy, then the co-treatment of Utt-B and Baf A1 should cause a surplus accumulation of LC3II in HepG2 cells, compared to the cells treated with either of the compounds, alone. Analysis of LC3 II expression in HepG2 cells in response to different concentrations of Baf A1 revealed that 10nM Baf A1 is enough for completely blocking autophagosome-lysosome fusion in HepG2 cells (**Figure 1E**). Interestingly, the co-treatment of Baf A1 with Utt-B caused a surplus accumulation of LC3II in HepG2 cells compared to treatment with either of the compounds alone, confirming that over-expression of LC3II in Utt-B-treated cells is not because of autophagic blockage, but due to enhanced autophagosome synthesis, demonstrating that Utt-B is a strong inducer of autophagy (**Figure 1F**).

To generate further proof for autophagy induction by Utt-B, we used Tf-LC3, an LC3 reporter construct tagged with red (mRFP) and green (GFP) fluorescence proteins, which can keep track of the autophagosome maturation process (19). These protein construct, in autophagosomes, emits both red and green fluorescence (mRFP^+^-GFP^+^), thus presenting yellow fluorescence in merged confocal images. However, when the autophagosomes mature to autophagolysosomes, the reporter protein emits only red fluorescence (mRFP^+^-GFP^-^) because of the quenching of GFP fluorescence in the acidic lysosomal environment of autophagolysosomes. We transiently transfected PtfLC3 to HepG2 cells, treated with Utt-B, and examined the fluorescence by confocal imaging. The number of autophagosomes and their maturation to autophagolysosomes were quantified by counting and comparing mRFP^+^-GFP^+^ and mRFP^+^-GFP^-^ punctae in control and Utt-B-treated wells. The data demonstrate that Utt-B-treated cells exhibit a significant increase in mRFP^+^-GFP^+^ (autophagosomes) along with mRFP^+^-GFP^-^ (autophagolysosomes), which implies Utt-B-induced formation of autophagosomes and their progression to autophagolysosomes (**Figures 1G, H**). Taken together, these results attest that Utt- B is an efficient inducer of autophagy.

### Uttroside B Induces ATG Proteins and Modulates Autophagy Regulating the AMPK-mTOR Signaling Axis

**Figure 2:**
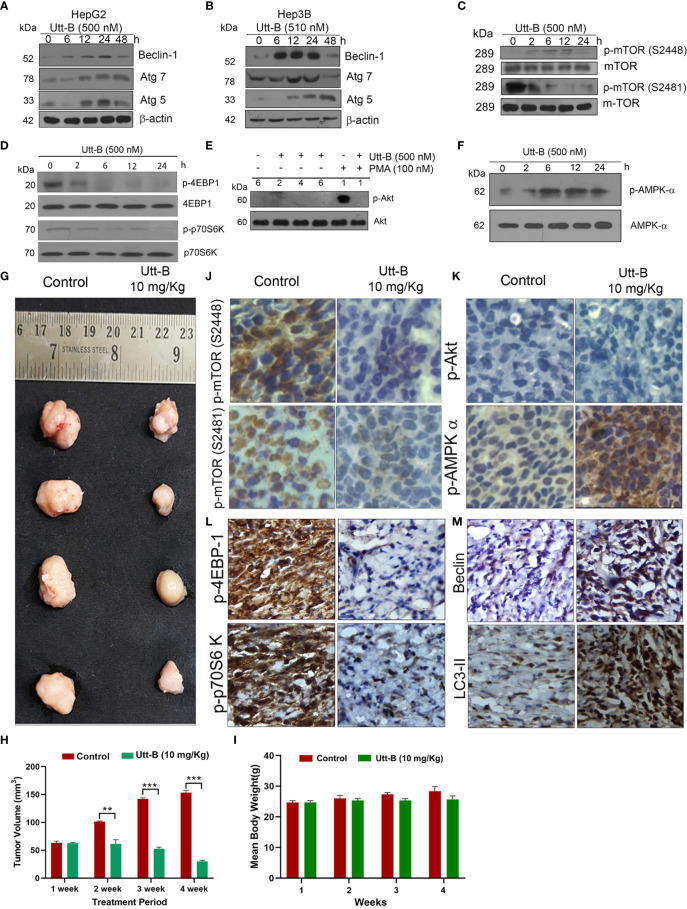
Utt-B activates autophagy-related proteins and modulates autophagy by regulating the AMPK-mTOR signaling axis. **(A)** Immunoblot analysis shows that Autophagy markers Beclin1, Atg7 and Atg5 are elevated up to 24h upon Utt-B treatment (500nM) and declines at 48h in HepG2 cells. **(B)** Beclin1, Atg7, Atg5 elevates up to 24h upon Utt-B treatment(500nM) and decline at 48h in Hep3B cells. **(C)** Utt-B treatment inhibits both p-mTOR (S2448) and p-mTOR (S2481). **(D)** Utt-B treatment significantly down-regulates p-70S6 Kinase and p-4EBP1, the downstream targets of mTOR. **(E)** Utt-B completely abolishes PMA-induced activation of Akt. **(F)** Utt-B phosphorylates AMPK alpha. **(G, H)** Utt-B significantly inhibits the growth of HepG2 xenografts in NOD-SCID mice. **(I)** Change in Body weight of mice in control and Utt-B treatment is non-significant throughout the period of experiment **(J–M)** Immunohistochemical analysis shows that Utt-B administration down-regulates the activation of p-mTOR (S2448), p-mTOR (S2481), p-70S6 Kinase, and p-4EBP1, with no change in p-Akt status and up-regulates the expression of p-AMPK-alpha and the autophagy markers, Beclin 1 and LC3. ** level of significance 2, *** level of significance 3.

Next, we evaluated the expression status of Beclin1, Atg5, and Atg7, the major proteins regulating the machinery of autophagy, in both Hep3B and HepG2 cells, in response to Utt-B. Beclin1 is a protein functioning in the initial assembly of the autophagosome, whereas Atg5 and Atg7 are proteins involved in the ubiquitin-like conjugative systems required for the proper development of autophagosomes. We found that Utt-B induces Beclin 1 in both HepG2 and Hep3B cells. In HepG2 cells, induction of Beclin1 starts from 12 h onwards and remains elevated up to 48 h, whereas the expression of Beclin1 in Hep3B cells was found to be increased at 24 h. Moreover, in both the cells, it tremendously escalates the expression of Atg7 and Atg5 from 12 h onwards. While Atg7 remains elevated up to 48 h after Utt-B treatment, the expression of Atg5 peaks at 24 h and declines at 48 h (**Figures 2A, B**). These data suggest that Utt-B induces a general increase in the expression of crucial proteins of autophagy machinery in liver cancer cells, independent of their HBV status. Transcriptional level alterations of the key autophagy related genes like LC3 and Beclin have been reported in different cancer cell lines, including HepG2, in response to various stimuli (20, 21). Hence post translational changes induced in liver cancer in response to Utt-B needs to be further evaluated.

Further, we analyzed the expression status of various signals involved in the regulation of autophagy in liver cancer cells, in response to Utt-B. mTOR plays a pivotal role in cell growth and metabolism of HCC and is up-regulated in 40-50% of HCC. Moreover, an up-regulation of mTOR is frequently observed in cholangiocarcinoma, the second most common primary cancer of the liver (22). It has been shown that Akt and AMPK pathways have a very strong role in regulating mTOR signaling. While the Akt pathway up-regulates mTOR signaling, the AMPK pathway does the reverse (23). The negative regulation of AMPK is known to block mTOR activation, thus relieving the suppression of autophagy exerted by mTOR, leading to the activation of autophagy (24). Hence, it is important to evaluate the expression status of mTOR, and downstream signaling along with Akt and AMPK pathways, in response to Utt-B treatment, as all these pathways are interrelated and play crucial roles in regulating liver cancer progression.

Hence, we studied the phosphorylation status of mTOR and mTOR target proteins, p70S6 Kinase and p4E-BP1 followed by that of Akt and AMPK, in Utt-B-treated HepG2 cells. Results demonstrated a time-dependent decrease in the phosphorylation of mTOR and its substrates p70S6 Kinase and p4E-BP1, whose phosphorylation can be considered as the readout of mTOR activity, which reached a minimum at 6 h and remained down-regulated up to 24 h following Utt-B treatment (**Figures 2C, D**). Concomitantly, AMPK phosphorylation was strongly up-regulated from 6h onwards and remained elevated up to 24 h following Utt-B treatment (**Figure 2F**), indicating a strong correlation between mTOR down-regulation and AMPK up-regulation. We have previously noted that Utt-B does not induce Akt phosphorylation in HepG2 cells (6). Now we checked whether it can down-regulate Akt phosphorylation induced by external stimuli such as Phorbol 12-Myristate 13-Acetate (PMA). It was very interesting to see that Utt-B completely abolished the PMA-induced activation of Akt (**Figure 2E**). These results strongly indicate that Utt-B induces autophagy in hepatic cancer cells, by modulating AMPK-mTOR signaling. We verified these results *in vivo*, in NOD-SCID mice bearing liver cancer xenografts, which manifested significant tumor reduction on Utt-B treatment (**Figures 2G, H**). **Figure 2I** clearly indicates that there is no significant change in the body weight of the control as well as Utt-B treated animals, throughout the period of experiment.

The tumor tissues were analyzed for the activation status of mTOR-AMPK-Akt signaling. Attesting the results of our *in vitro* study, we observed a significant down-regulation in the phosphorylation status of mTOR, p70S6 Kinase, p4E-BP-1 (**Figures 2J, L**). As observed in our *in vitro* studies we did not find Akt phosphorylation, either in the control or Utt-B treated tumors Similarly, attesting our *in vitro* observations, strong phosphorylation of AMPK α (**Figure 2K**) was noted in the xenografts, treated with Utt- B. Increased expression of autophagy markers Beclin 1 and LC3-II also observed in the Utt-B treated tumors (**Figure 2M**).

### Inhibition of Autophagy Enhances Uttroside B-Induced Apoptosis and Cell Death in Hepatic Cancer Cells

**Figure 3:**
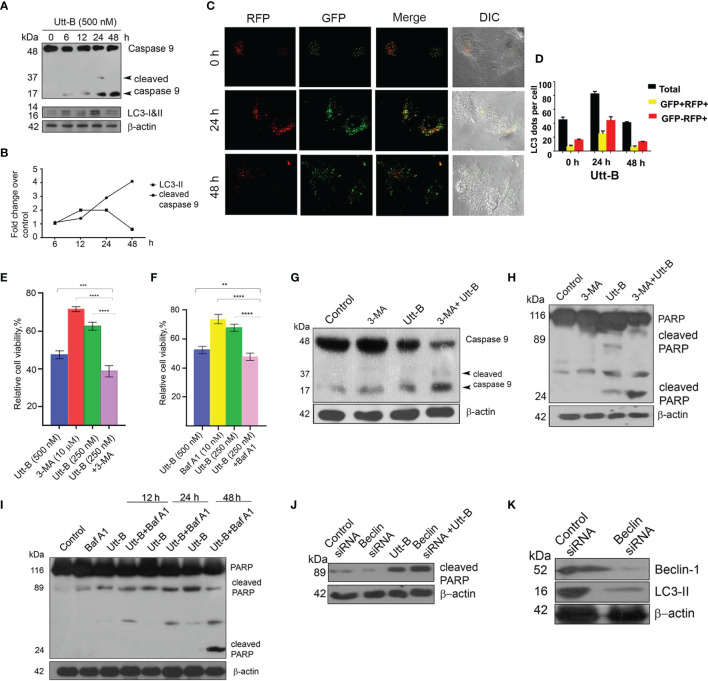
Inhibition of autophagy enhances Utt-B-mediated apoptotic program **(A, B)** Utt-B treatment induces the cleavage of caspase 9 at 24 h and increases further up to 48 h, and accumulation of LC3-II at 12 h and is maintained up to 24 h, followed by its degradation by 48 h. **(C, D)** RFP-GFP-LC3 tagged assay shows more mRFP^+^-GFP punctae in HepG2 cells, up to 24 h followed by a significant reduction in the number of punctae at 48 h. **(E, F)** Co-treatment with both the inhibitors 3-MA and Baf A1enhances Utt-B-mediated cytotoxicity in HepG2 cells. **(G–I)** Co-treatment of autophagy inhibitors with Utt-B induces the Cleavage of Caspase 9 and PARP. **(J, K)** Beclin SiRNA silenced-HepG2 cells exhibit increased expression of cleaved PARP. The error bars represent the Standard Deviation. One-way ANOVA followed by Tukey’s post hoc t test analysis was used for statistical comparison between different groups. ***P ≤ 0.001; **P ≤ 0.01. Symbol asterisk (*) represents statistical significance between control and treatment groups **(E, F)**.

The interrelation between autophagy and apoptosis in response to chemotherapeutic stress has a crucial role in determining tumor cell death. Hence, we attempted to analyze the extent of the influence of autophagy on Utt-B-induced apoptosis in HepG2 cells. First, we analyzed the timings at which Utt-B induces apoptosis and autophagy, by studying the activation status of the initiator caspase 9 and the expression of LC3II at different time points of Utt-B treatment. The cleavage of caspase 9 (p17) begins at 24 h and increases further up to 48 h, whereas the accumulation of LC3-II peaks at 12 h and is maintained up to 24 h, followed by its degradation by 48 h (**Figures 3A, B**). To further confirm the dynamics of autophagy induction due to Utt-B treatment, Tf-LC3 transfected HepG2 cells were treated with Utt-B and quantified the fluorescent punctae in the cells at different time points. It was very interesting to see that Utt-B treatment escalates the number of total GFP punctae, mRFP^+^-GFP^+^ punctae, and mRFP^+^-GFP punctae in HepG2 cells, up to 24 h and, there is a significant reduction in the number of all the three at 48 h (**Figures 3C, D**). Both these experiments reveal that there is an autophagy-activated phase, up to 24 h, prior to apoptosis, in Utt-B -induced cell death program in HepG2 cells. So, we inhibited autophagy with the pharmacological inhibitors of autophagy, 3-methyladenine (3-MA), and Baf A1 and asked whether Utt-B induced autophagy is complementing or contradicting the apoptosis and cell death program induced by the compound in HepG2 cells. Interestingly, inhibition of autophagy by both 3-MA and Baf A1 significantly enhanced Utt-B-induced cytotoxicity (p values;0.005) indicating that Utt-B-induced autophagy is blocking its apoptotic program. (**Figures 3E, F**). Co-treatment of 3-MA and Baf A1enhance the cytotoxicity of 250 nM Utt-B in HepG2 cells from 25% to 51% and from 31% to 52% respectively, which is equivalent to the cytotoxicity induced by 500 nM Utt-B, indicating that Utt-B-induced autophagy is blocking its apoptotic program. This observation was confirmed by studying the activation status of caspase 9 and PARP, the key players of the Utt-B-induced apoptotic program, in presence of 3-MA and Baf A1. Co-treatment with both the inhibitors enhances Utt-B -induced cleavage of PARP (**Figures 3G, H**) confirming that Utt-B -induced autophagic flux contradicts its efficacy in inducing apoptosis and inhibition of these autophagic signals by an external agent can enhance the therapeutic potential of the compound. (**Figure 3I**). We re-confirmed these postulations, by genetically inhibiting autophagy by silencing Beclin1 (**Figure 3J**). As expected, Utt-B induced a strong enhancement in PARP cleavage in Beclin1-silenced HepG2 cells, compared to the control HepG2 cells (**Figure 3K**) underscoring that autophagy induced by Utt-B is pro-survival in nature and inhibition of this autophagy could be utilized as a strategy to enhance the anti-tumor efficacy of Utt-B.

### Co-Treatment of Chloroquine With Uttroside B Enhances The Anti-Cancer Efficacy of Uttroside B Against Hepatic Cancer Cells

**Figure 4:**
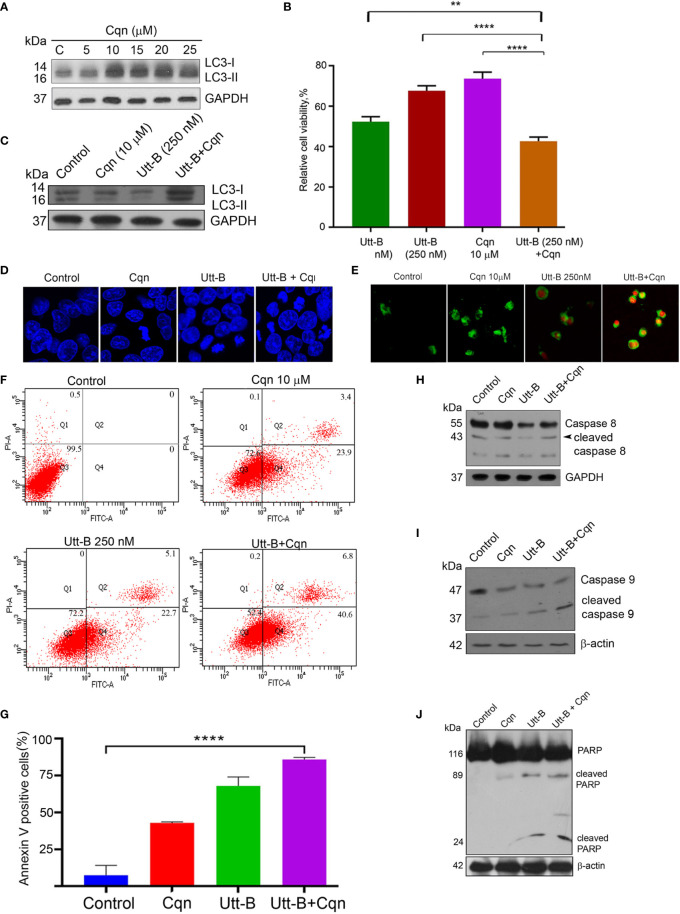
Co-treatment of Cqn with Utt-B enhances the anti-cancer efficacy of Utt-B, *in vitro.* **(A)** 10 µM Cqn blocks autophagy in HepG2 cells as shown by the accumulation of LC3-II. **(B)** Co-treatment of sub-toxic concentration of Cqn with Utt-B enhances the cytotoxicity of Utt-B, in HepG2 cells. **(C)** Co-treatment of a sub-toxic concentration of Cqn with Utt-B enhances the accumulation of LC3-II. **(D)** DAPI staining reveals that co-treatment of Cqn and Utt-B enhances the nuclear condensation of HepG2 cells. **(E)** Annexin-PI staining indicates an enhancement in apoptosis in the wells treated with the combination. **(F, G)** Annexin-PI flow cytometric analysis shows an increase in apoptotic cells in the co-treated cells. **(H–J)** Western blot analysis shows an enhancement in cleavage of caspase 8, caspase 9, and PARP in HepG2 cells treated with the combination of Cqn and Utt-B. ** level of significance 2, **** level of significance 4. The error bars represent the Standard Deviation. One-way ANOVA followed by Tukey’s post hoc t test analysis was used for statistical comparison between different groups. ****P ≤ 0.0001; **P ≤ 0.01, Symbol asterisk (*) represents statistical significance between control and treatment groups **(B, G)**.

Next, we investigated whether co-treatment of the antimalarial drug, Cqn, which is a well-known inhibitor of autophagy. We can enhance the anticancer efficacy of Utt-B against hepatic cancer cells. Cqn inhibits autophagy by blocking the fusion of autophagosomes with lysosomes and hence, cause the accumulation of autophagosomes and an increase in LC3II. The concentration of Cqn required to fully block autophagosome-lysosome fusion was determined by analyzing the accumulation of LC3II in HepG2 cells treated with different concentrations of chloroquine. LC3-II formation peaked at 10 µM Cqn, without further concentration-dependent change, suggesting that 10 µM Cqn is enough to completely block autophagosome-lysosome fusion. (**Figure 4A**). Hence, we used this concentration for blocking autophagy induced by Utt- B. IC 50 of Cqn in HepG2 was estimated as 20 µM (**Figure S1A**). It was interesting to see that while 10 µM Cqn and 250 nM Utt-B induced 26.34% and 29.96% cytotoxicity, respectively, in HepG2 cells, a combination of these two induced 52.23% cytotoxicity, which is equivalent to that induced by 500 nM Utt-B alone, in MTT assay (**Figure 4B**). Moreover, co-treatment of both the compounds caused an enhanced accumulation of LC3II in HepG2 cells compared to the cells treated with either of them alone; again proving that Utt-B is an autophagy inducer (**Figure 4C**). In wound healing assay, the wound closure was much slower in the wells treated with the combination compared to that treated with either of the compounds (**Figure S1B**). The results from the clonogenic assay were also in concordance with that of the MTT assay. While the combination induces a 76.56% reduction in the clonogenicity of HepG2 cells, 10 μM Cqn and 250 nM Utt-B could individually induce only 42% and 51% reduction respectively (**Figures S1C, D**). DAPI staining for assessing nuclear condensation shows an increase in the number of condensed nuclei (**Figure 4D** and **Figure S1F**) and Annexin V FITC staining for apoptosis shows a greater number of annexin/PI-stained cells (**Figure 4E**) in the wells treated with the combination and also by the FACS analysis (**Figures 4F, G**). Efficacy of the combination in enhancing Utt-B induced apoptotic program is confirmed by the cleavage of caspase 8 (**Figure 4H**) caspase 9 (**Figure 4I**) and PARP (**Figure 4J**). However, neither Utt-B nor Cqn produced significant cell cycle arrest either alone or in combination (**Figure S1E**). Taken together, these results demonstrate that chloroquine can strongly enhance the anticancer efficacy of Utt-B by inhibiting the survival autophagic flux induced by the compound.

### The Combination of Chloroquine and Uttroside B Is Pharmacologically Safe in Mice

**Figure 5:**
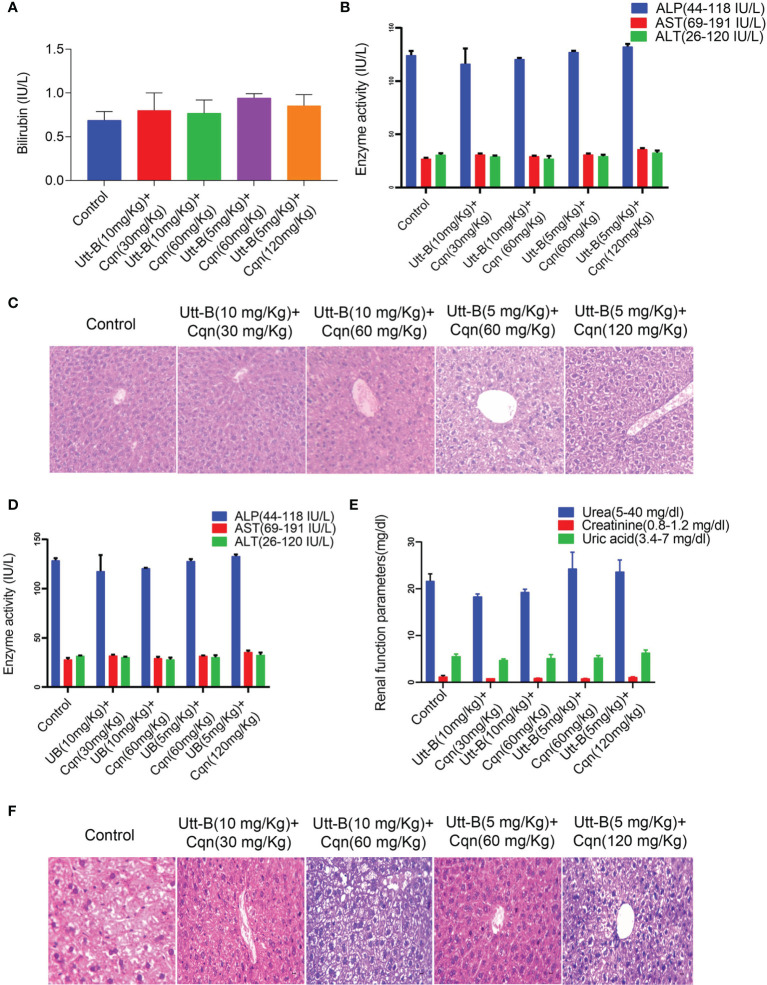
Combination of Cqn and Utt-B is pharmacologically safe in *Swiss Albino* mice. **(A, B)** Acute toxicity analysis of Bilirubin and liver enzymes such as AST, ALT, and ALP values show all the combinations are within the range. **(C)** Hematoxylin and eosin staining in liver tissues of control and treatment groups of acute toxicity study **(D, E)** Liver and renal function parameters of Sub-chronic toxicity analysis of the combination **(F)** Histopathology of liver tissues from sub-chronic toxicity analysis.

As our *in vitro* studies demonstrate that co-treatment of sub-toxic concentrations of Cqn and Utt-B can tremendously improve its anticancer potential against HCC, we evaluated the biological safety of different combinations of Cqn and Utt-B, by conducting acute and sub-chronic toxicity studies in Swiss albino mice, before going for a preclinical evaluation of the combination. For acute toxicity studies, the test groups were treated with different doses of Cqn (0, 30, 60 &120, mg/kg body weight, n=6 for each dose) and different combinations of Utt-B and Cqn (10 + 30, 10 + 60, 5 + 60, and 5 + 120 mg/kg body weight, n=6 for each combination). The biological safety of Utt-B has already been confirmed in our previous study (6). Similarly, for sub-chronic toxicity studies also, the test groups were treated with the same doses of Cqn and the combinations (**Figure S2A**). At the end of the experiment, the mice in the acute and sub-chronic study schedules were euthanized after 14 days and 90 days, respectively. The liver, kidney, and spleen tissues were analyzed for histopathology using H&E staining and the serum samples were used to perform Liver Function Test (LFT) and Renal Function Test (RFT). Up to 120 mg/kg of Cqn treatment was safe, both in acute and sub chronic toxicity studies (**Figures S2B–G**). Histopathological analysis reveals that the doses, 5 + 60 mg/kg, corresponding to the combination in the *in vitro* study, i.e., (250 nM Utt-B+10µM Cqn), are biologically safe. In the 120 mg/kg Cqn group, the liver shows mild regeneration, though there was no hepatitis/necrosis/fatty change. In 10 + 60 and 5 + 120 combinations liver shows mild degenerative changes in hepatocytes. (**Figures 5C, F**). Though some of the biochemical parameters used in LFT and RFT are altered in groups that received the combination (5/120 mg/kg & 10/120 mg/kg), none of them exceeds the normal range, and all the values are non-significant among groups suggesting the pharmacological safety of the combination (**Figures 5A, B, D, E**). Taken together, both acute and chronic toxicity studies confirm that combined use of subtoxic doses of Cqn and Utt- B is safe for *in vivo* treatments.

### Co-Treatment of Chloroquine Enhances the Antitumor Efficacy of Uttroside B Against HCC

**Figure 6:**
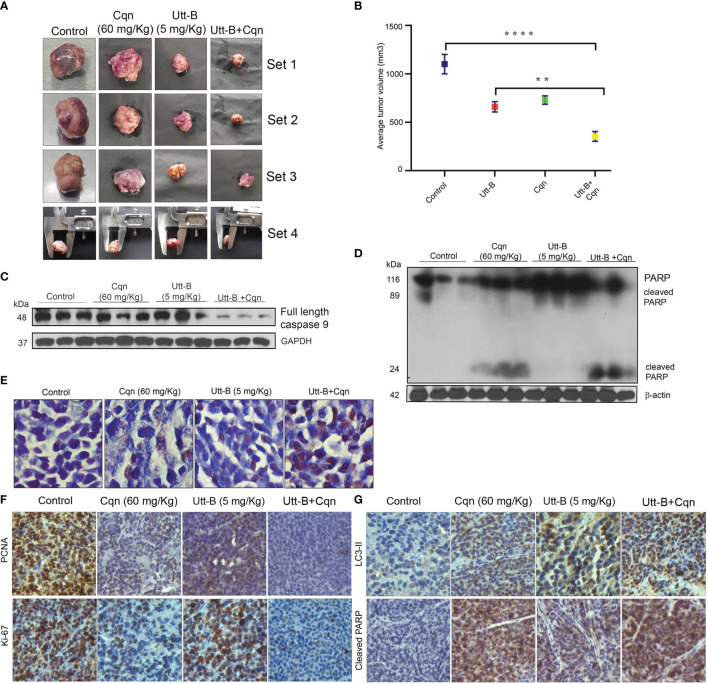
Co-treatment of Cqn enhances the antitumor efficacy of Utt-B against HCC, *in* NOD-SCID mice bearing HepG2 xenografts.** (A, B)** Tumor images and Graphical representation of final tumor volume of different groups. **(C, D)** Western blot analysis demonstrating enhanced cleavage of caspase 9 motherband and PARP in the group co-treated with Cqn and Utt-B, compared to control and individual treatments. **(E)** Increase in TUNEL-positive cells in the group co-treated with Cqn and Utt-B, confirming enhancement in apoptosis **(F, G)** Immunohistochemical analysis of nuclear proliferation markers PCNA, ki67 and the autophagy marker LC3-II and Apoptotic marker Cleaved PARP. ** level of significance 2, **** level of significance 4. Data represent three independent sets of experiments. The error bars represent the Standard Deviation. Statistical significance was analysed by Student’s t test. ****P ≤ 0.0001; **P ≤ 0.01. Symbol asterisk (*) represents statistical significance between control and treatment groups **(B)**.

Cqn is known to block the pro-survival autophagy induced by different chemotherapeutic agents, thereby enhancing their antitumor potential (25). As our *in vitro* studies revealed that co-treatment of subtoxic concentration of Cqn doubles the anti-tumor efficacy of sub-optimal dose of Utt-B, we examined the efficacy of the combination, *in vivo*, in NOD-SCID mice bearing HepG2 xenografts. The control group received vehicle (PBS) and the other groups received Utt-B (5 mg/kg), Cqn (60 mg/kg), or the combination of Cqn (60 mg/kg) and Utt-B (5 mg/kg), intraperitoneally (**Figure S3A**) We found that the mice that received the combination exhibit statistically significant tumor regression (3.3fold, p value=0.005) compared to those received either Cqn (1.4-Fold) or Utt-B (1.6-Fold) alone (**Figures 6A, B** and **Figure S3C**).

The tumor tissues collected from mice that received the combination of Cqn and Utt-B exhibited strong cleavage of caspase 9 mother band and PARP (**Figures 6C, D**). Histopathological investigation of the tumor samples showed significantly higher necrosis in the group treated with the combination, compared to other groups (**Figure S3B**). TUNEL staining performed in tumor tissues collected from the different groups demonstrates a significant increase in the number of apoptotic cells in the tumor tissues of mice treated with the combination. However, we did not observe significant apoptotic cells in the tumor tissues collected from mice that received either Cqn or Utt-B alone (**Figure 6E**). Immunohistochemical analysis shows significant down-regulation of the proliferative marker, ki67, and up-regulation of cleaved PARP, indicative of apoptosis, in the group treated with the combination Expression status of the Autophagy marker, Beclin 1 was high in Utt-B treated group, and significantly less in all other groups. The tumor sections from the Cqn-treated group and the combination group displayed over-expression of LC3 II, confirming the accumulation of autophagosome caused by the blockage of autophagosome-lysosome fusion by Cqn (**Figures 6F, G**). Taken together, these results suggest that the antimalarial drug, Cqn, when repurposed as an autophagy inhibitor, enhances Utt-B-induced apoptotic program, thereby improving its chemotherapeutic efficacy against HCC.

## Discussion

The present study demonstrates that Utt-B, a saponin recently patented from our lab as a potent anti-HCC drug, and is on the track of clinical trials for hepatic cancer treatment, based on our findings, induces autophagy, in addition to apoptosis, in hepatic cancer cells. Autophagy induced in response to chemotherapeutic agents is mainly considered as a factor capable of influencing cell death in a wide range of tumors, including hepatic cancer (26, 27). Depending upon the nature of the chemotherapeutic strategy, autophagy can either act as a pro-death mechanism by hyper digesting the cellular components or as a pro-survival factor resisting cell death (28). The efficacy of chemotherapeutic drugs capable of inducing pro-survival autophagy in hepatic cancer cells can be enhanced by blocking autophagy. For example, the chemotherapeutic potential of sorafenib in hepatic cancer cells, both *in vivo* and *in vitro*, can be enhanced by inhibiting autophagy (29, 30). Similarly, the chemotherapeutic potential of bevacizumab against hepatic cancer cells has also been increased by blocking autophagy (31). Our results demonstrate that inhibition of autophagy either through inhibiting Beclin1 expression or through pharmacological agents has enhanced Utt-B-induced cell death and apoptosis in hepatic cancer cells.

Our attempt to study the role of autophagy in cell death not only reveals the pro-survival facet of Utt-B-induced autophagy but also disclose the interesting dynamics involved in the autophagy-apoptosis interplay in hepatic cancer cells during Utt-B-induced cell death. We found that there was an initial autophagy dominant phase in Utt-B-treated hepatic cancer cells with no apoptosis initiation, and this phase was followed by a late apoptosis activating phase, where autophagic signals were found diminishing. The initial autophagy in response to Utt-B treatment is likely to be a futile survival attempt of hepatic cancer cells, which delay the apoptotic events induced by Utt-B treatment. There are widely known general strategies utilized by tumor cells through autophagy for delaying apoptosis in tumor cells. For instance, selective degradation of damaged mitochondria in the cytosol, due to autophagy, delays the release of cytochrome c from it, thereby delaying the initiation of apoptosis. Moreover, autophagy is capable of selective degradation of components such as caspase 8, which are involved in the apoptosis mechanism, leading to the delay in apoptosis (32).

We have shown that Utt-B blocks mTOR signaling and activate AMPK signaling in hepatic cancer cells. Both mTOR inhibition and AMPK activation are prominent signaling mechanisms regulating autophagy in tumor cells (33). The mTOR complex 1 (mTORC1), a functional component of mTOR, negatively modulates autophagy by phosphorylating and inhibiting ULK 1, a protein involved in autophagy initiating machinery (34). Inhibition of mTOR blocks the inhibitory phosphorylation of ULK1 and thus activates autophagy. Similarly, AMPK signaling acts antagonistic to mTOR in modulating autophagy. Activated AMPK could positively regulate autophagy through enhancing pro-autophagic functions of ULK 1 or indirectly by activating TSC1, a modulator protein complex that inhibits mTOR (35, 36). Since Utt-B activates AMPK and blocks mTOR signaling reciprocally, it may be assumed that Utt-B-induced autophagy is regulated by either of these pathways; ie, AMPK activation or blockage of mTOR.

Our study also reveals that co-treatment with a subtoxic concentration of Cqn, a well-known inhibitor of autophagy, significantly enhances the anti-tumor potential of Utt-B against HCC-xenografts developed in NOD/SCID mice. We found that the combination of Cqn and Utt-B is pharmacologically safe *in vivo*. Cqn is considered a pharmacologically safe and clinically useful option for autophagic inhibition. It has been used in a range of clinical trials for blocking autophagy in tumors, including human trials designed to enhance the chemotherapeutic potential of sunitinib in HCC (37). Our results demonstrating the enhancement of chemotherapeutic potential of Utt-B in combination with the autophagic inhibitor, Cqn, reveal that the easiest option for enhancing the chemotherapeutic impact of Utt-B is to negatively modulate autophagy. However, the present study was conducted in NOD/SCID mice, which lack mature B and T cells, but possess residual NK cells and lymphocytes (38). Studies conducted in DBA2J mice have demonstrated that autophagy plays a differential role in T-cell functions (39). Hence we have initiated further *in vivo* studies in DBA/2J mice to assess the efficacy of Utt-B, using an environmental carcinogenesis model.

**Figure 7:**
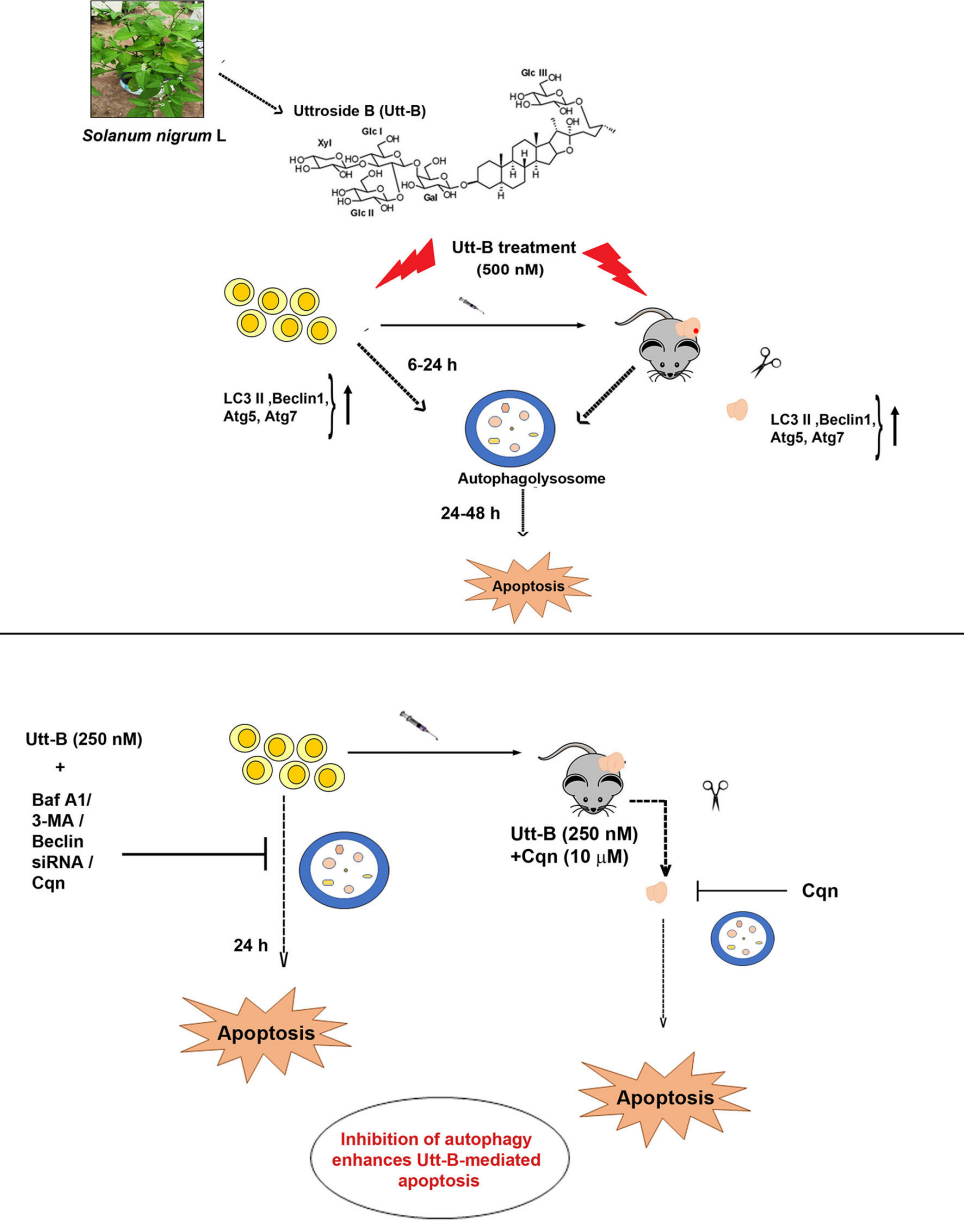
Inhibition of Utt-B-induced autophagy improves the therapeutic efficacy of Utt-B against HCC. Utt-B, a furostanol glycoside, isolated from the methanolic extract of *Solanum nigrum* L, is found to be an inducer of autophagy as evidenced by the up-regulation of autophagy markers such as LC3 II, Beclin 1, atg5, and atg7, both *in vitro* and *in vivo*. At *in vitro* conditions, autophagy peaks from 6-24h, followed by apoptosis. Inhibition of Utt-B induced autophagy, by pharmacological autophagy inhibitors such as Baf A1, 3-MA, Cqn, and biological inhibitor, Beclin siRNA enhances Utt-B mediated apoptosis, *in vitro*. Inhibition of Utt-B-induced autophagy by sub-toxic concentrations of Cqn enhances the chemotherapeutic potential of Utt-B, *in vivo*.

In summary, our study demonstrates that Utt-B induces pro-survival autophagy, which is capable of delaying its apoptosis program in hepatic cancer cells. Moreover, our results clearly depict that Cqn, a drug already being used in the clinics against malaria, if repurposed as an autophagy inhibitor and used in a combinatorial regimen with Utt-B, can improve the therapeutic efficacy of Utt-B against HCC. Our results have been summarized in (**Figure 7**) and we hope these findings will help to design future strategies based on regimens for inhibiting Utt-B-induced autophagic response for improving its chemotherapeutic efficacy against HCC.

## Data Availability Statement

The original contributions presented in the study are included in the article/**Supplementary Material**. Further inquiries can be directed to the corresponding author.

## Ethics Statement

Studies involving experiments with animals were conducted in accordance with institution guidelines, under the approval from Institutional Animal Ethics Committee, Rajiv Gandhi Centre for Biotechnology. (CPCSEA Number:326/GO/ReBiBt/S/2001/CPCSEA).

## Author Contributions

LN: Investigation, Literature search, Study design, Data collection, Data analysis, Data interpretation. MS: Investigation, Literature search, Figures, Formal analysis, Study design, Data collection, Data analysis, Data interpretation, writing. VV: Literature search, Data Analysis, Conceptualization. AT: Resources. HN: Figures, Project administration. SA: Data Collection. SUA: Data collection. TR: Figures. CK: Data collection. KK: Review & editing, SS: Data analysis, Data interpretation. RL: Resources. SP: Review & editing. RT: Review & editing. NI: Review & editing. RA: Conceptualization, Supervision, Writing- review & editing. All authors contributed to the article and approved the submitted version.

## Funding

Financial support from DST-SERB (EMR/2016/00644). The funders had no role in study design, data collection, data analysis, interpretation, and writing of the manuscript.

## Conflict of Interest

The authors declare that the research was conducted in the absence of any commercial or financial relationships that could be construed as a potential conflict of interest.

## Publisher’s Note

All claims expressed in this article are solely those of the authors and do not necessarily represent those of their affiliated organizations, or those of the publisher, the editors and the reviewers. Any product that may be evaluated in this article, or claim that may be made by its manufacturer, is not guaranteed or endorsed by the publisher.

## Abbreviations

Utt-B, Uttroside B; Cqn, Chloroquine; HCC, Hepatocellular carcinoma; Baf A1, Bafilomycin A1; 3-MA, 3- Methyl Adenine; mTOR, mammalian inhibitor of rapamycin; TUNEL, Terminal deoxy-nucleotidyl transferase-mediated nick end labeling; PMA, Phorbol 12-Myristate 13-Acetate; H&E staining, Haematoxylin and Eosin staining; h, hour; LFT, Liver Function Test; RFT, Renal Function Test; p-Akt, phospho-Protein Kinase B; p70S6K, phospho- Ribosomal protein S6 kinase; p4EBP1, phospho- Eukaryotic translation initiation factor 4E – binding protein 1; AMPK, AMP- activated protein kinase; LC3, Microtubule associated protein 1A/1B- light chain 3-; ATG, Autophagy related gene; PARP, Poly adenosine diphosphate-ribose polymerase; ki67, Marker of proliferation ki67; PCNA, Proliferating cell nuclear antigen; PBS, Phosphate buffered saline;IP, Intra-peritoneal; TSC-1, Tuberous sclerosis 1; ULK 1-1,Unc-51 like autophagy activating kinase 1; GAPDH, Glyceraldehyde 3-phosphate dehydrogenase; FACS, Fluorescence associated Cell sorting; FBS, Foetal bovine serum; GFP, Green fluorescence protein; RFP, Red fluorescence protein; HBV, Hepatitis B Virus; FACS, Fluorescence Associated Cell Sorting.
