# Corrigendum: Blockade of uttroside B-induced autophagic pro-survival signals augments its chemotherapeutic efficacy against hepatocellular carcinoma

**DOI:** 10.3389/fonc.2022.991401

**Published:** 2022-08-25

**Authors:** Lekshmi R. Nath, Mundanattu Swetha, Vinod Vijayakurup, Arun Kumar Thangarasu, Nair Hariprasad Haritha, Anwar Shabna, Sreekumar U. Aiswarya, Tennyson P. Rayginia, C. K. Keerthana, Kalishwaralal Kalimuthu, Sankar Sundaram, Ravi Shankar Lankalapalli, Sreekumar Pillai, Rheal Towner, Noah Isakov, Ruby John Anto

**Affiliations:** ^1^ Division of Cancer Research, Rajiv Gandhi Centre for Biotechnology, Thiruvananthapuram, India; ^2^ Chemical Sciences and Technology Division, Council for Scientific and Industrial Research (CSIR)-National Institute for Interdisciplinary Science and Technology, Thiruvananthapuram, India; ^3^ Academy of Scientific & Innovative Research (AcSIR), Ghaziabad, India; ^4^ Department of Biotechnology, University of Calicut, Malappuram, India; ^5^ Department of Pathology, Government Medical College, Kottayam, India; ^6^ Department of Surgical Oncology, Jubilee Mission Medical College and Research Institute, Thrissur, India; ^7^ Department of Pathology and Pharmaceutical Sciences, University of Oklahoma Health Sciences Center, Oklahoma, United States; ^8^ The Shraga Segal Department of Microbiology, Immunology, and Genetics, Ben-Gurion University of the Negev, Beer Sheva, Israel

**Keywords:** autophagy, chloroquine, apoptosis, uttroside B, hepatocellular carcinoma, chemoprevention

**Incorrect Supplementary Material**

In the published article, there was an error in **Supplementary Figure 1B**. A Duplicate image was given in Wound healing assay images of 0h Cqn (10µM) and Utt-B (250nM). The correct material statement appears below. There is no change in the wordings of the manuscript.

The authors apologize for this error and state that this does not change the scientific conclusions of the article in any way. The original article has been updated.

## Publisher’s note

All claims expressed in this article are solely those of the authors and do not necessarily represent those of their affiliated organizations, or those of the publisher, the editors and the reviewers. Any product that may be evaluated in this article, or claim that may be made by its manufacturer, is not guaranteed or endorsed by the publisher.

