# Supplementary material for: Blockade of Uttroside B-Induced Autophagic Pro-Survival Signals Augments Its Chemotherapeutic Efficacy Against Hepatocellular Carcinoma
Source: Front Oncol. 2022 Feb 8;12:812598. doi: 10.3389/fonc.2022.812598 (PMC8861526; doi:10.3389/fonc.2022.812598)
Supplement: Supplementary file 1 [file DataSheet_1.pdf]

Supplementary Material

1. Supplementary Figures

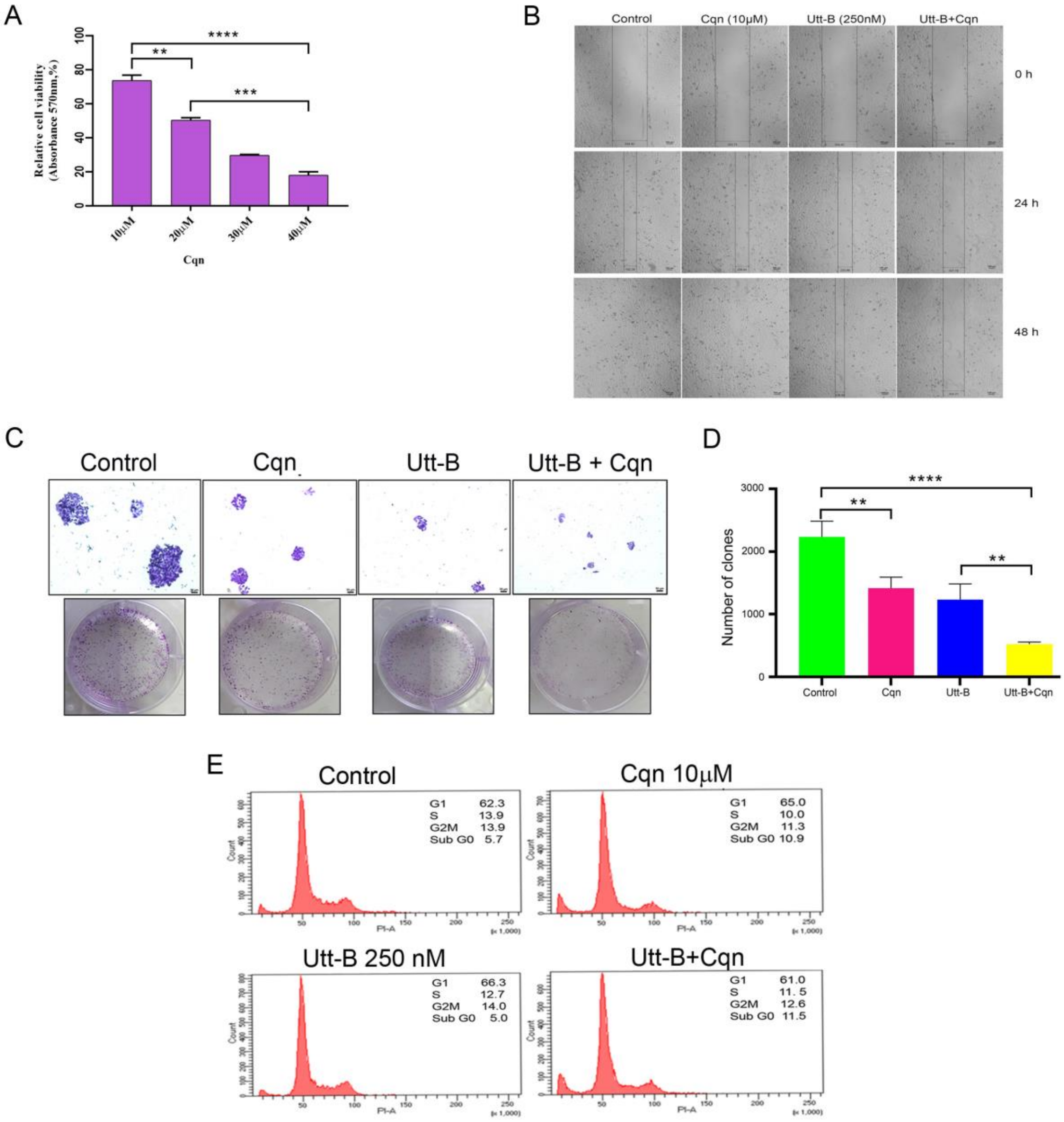

Supplementary Figure 1. Co-treatment of Cqn with Utt-B enhances the therapeutic efficacy of Utt-B against HCC, *in vitro* (A) IC 50 concentration of Cqn is 20 µM in HepG2 cells, as assessed by MTT Assay. (B) Wound healing assay shows that wound closure is much slower in co-treatment in comparison with that of individual treatments. (C, D) Clonogenic assay shows a decrease in the number and size of the colonies in the well co-treated with Cqn and Utt-B. (E). Utt-B and Cqn, either alone or in combination do not produce significant cell cycle arrest.

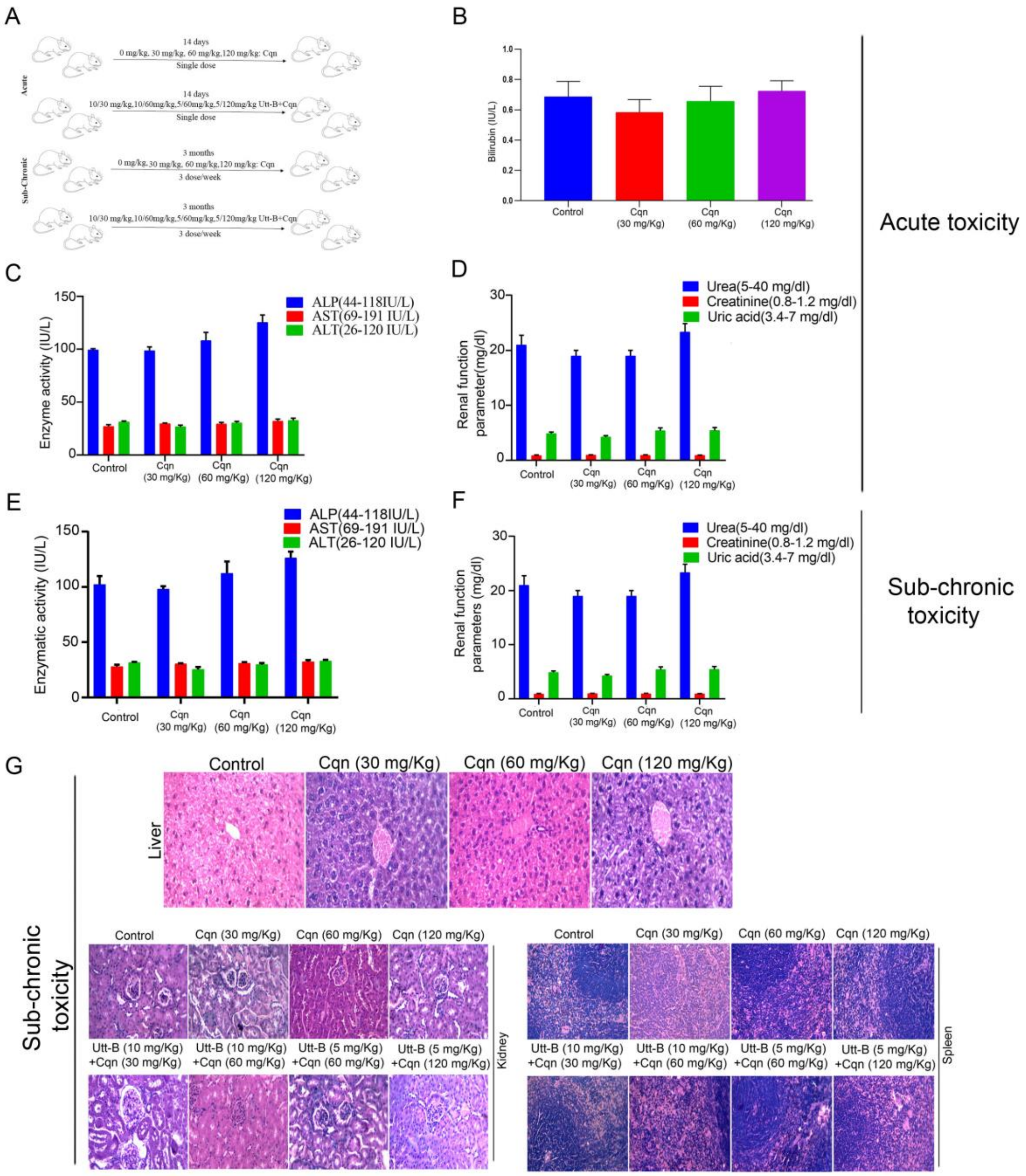

Supplementary Figure 2. Acute and subchronic toxicity analysis of Cqn and Utt-B in Swiss Albino mice. (A) Schematic diagram of Toxicity analysis of Cqn and combination of Cqn with Utt-B. (B, C, D) Bilirubin, Liver, and renal function parameters of the groups treated with different concentrations of Cqn in the Acute toxicity study. (E, F) Liver and renal function parameters of the groups treated with different concentrations of Cqn in the sub-chronic toxicity study. (G). Histopathology of liver tissues of groups treated with different concentrations of Cqn, histopathology of kidney and spleen of groups treated with different concentrations of either Cqn or combination of Utt-B and Cqn, in the sub-chronic toxicity study.

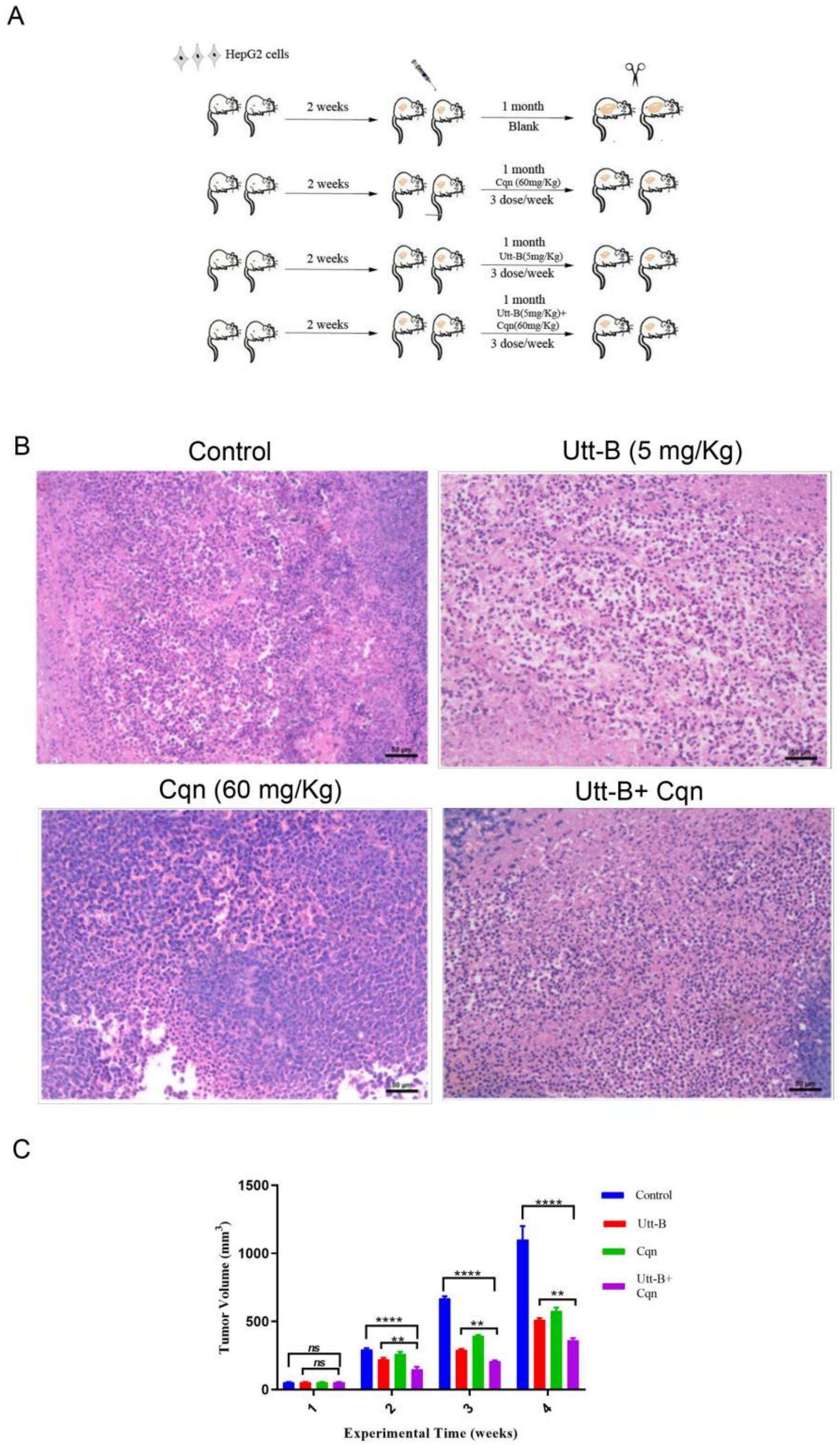

Supplementary Figure 3. **Co-treatment of Cqn enhances the antitumor efficacy of Utt-B against HCC, *in vivo*** (A) Schematic diagram of the anti-tumor study conducted for evaluating the efficacy of Cqn in enhancing the chemotherapeutic potential of Utt-B, using NOD-SCID mice bearing HepG2 xenografts. (B) Histopathological analysis of tumor tissues of anti-tumor study. (C) Graphical representation of weekly measurement of the tumor volume in the control and treatment groups, either alone or in combination.
