# Supplementary material for: Blockade of Uttroside B-Induced Autophagic Pro-Survival Signals Augments Its Chemotherapeutic Efficacy Against Hepatocellular Carcinoma
Source: Front Oncol. 2022 Feb 8;12:812598. doi: 10.3389/fonc.2022.812598 (PMC8861526; doi:10.3389/fonc.2022.812598)
Supplement: Supplementary file 2 [file DataSheet_2.pdf]

Supplementary Table 1

Figure 1					
Utt-B (500 nM)					
**Figure 1 C**	0 h	6 h	12 h	24 h	48 h
LC3-II	1.00	2.60	3.59	3.17	1.82
**Figure 1 D**	0 h	6 h	12 h	24 h	48 h
LC3-II	1.00	2.32	2.96	3.48	2.25

	Baf A1 (nM)			
**Figure 1 E**	0 nM	1 nM	10 nM	100 nM
LC3-II	1.00	3.95	6.08	6.12
**Figure 1 F**	C	Baf A1	Utt-B	Baf A1+ Utt-B
LC3-II	1.00	11.95	5.00	19.14

Figure 2					
Utt-B (500 nM)					
**Figure 2 A**	0 h	6 h	12 h	24 h	48 h
Beclin-1	1.00	3.18	5.19	8.27	3.06
Atg 7	1.00	0.77	2.36	2.85	3.92
Atg 5	1.00	0.38	2.65	3.69	1.77
**Figure 2 B**	0 h	6 h	12 h	24 h	48 h
Beclin-1	1.00	5.27	5.55	5.21	1.57
Atg 7	1.00	0.68	1.36	2.05	0.12
Atg 5	1.00	0.08	3.99	11.71	32.03
**Figure 2 C**	0 h	2 h	6 h	12 h	24 h
p-mTOR (S2448)	1.00	1.42	2.16	2.19	0.60
p-mTOR	1.00	0.37	0.12	0.05	0.08

(S2481)					
**Figure 2 D**	0 h	2 h	6 h	12 h	24 h
p-4EBP-1	1.00	0.40	0.35	0.33	0.32
p-p70S6 K	1.00	0.93	0.29	0.33	0.22
**Figure 2 F**	0 h	2 h	6 h	12 h	24 h
p-AMPKα	1.00	0.38	3.32	4.37	4.06

**Figure 2 E**	6 h (C)	2 h (Utt-B)	4 h (Utt-B)	6 h (Utt-B)	1 h (PMA)	1 h (Utt-B+ PMA)
p-Akt	1.00	2.95	0.58	0.35	43.30	0.34

**Figure 3**
Utt-B (500 nM)

**Figure 3A**	0 h	6 h	12 h	24 h	48 h
Full length Caspase 9	1.00	1.14	1.17	0.82	1.05
Cleaved casapase 9(p37)	1.00	0.98	1.02	9.57	0.93
Cleaved casapase 9(p17)	1.00	2.93	6.27	15.78	18.73
LC3-II	1.00	1.03	1.42	1.24	0.40

**Figure 3 G**	Control	3-MA	Utt-B	3-MA+ Utt-B
Full length Caspase 9	1.00	1.19	0.51	0.26
Cleaved casapase 9(p37)	1.00	3.24	1.68	18.84
**Figure 3 H**	Control	3-MA	Utt-B	3-MA+ Utt-B
PARP	1.00	0.72	0.41	0.99
Cleaved PARP (p24)	1.00	0.92	2.83	25.02

**Figure 3 J**	Control siRNA	Beclin siRNA	Utt-B	Beclin siRNA+ Utt-B
Cleaved PARP	1.00	0.52	5.60	6.64

**Figure 3 I**	Control	Baf A1	Utt-B 12h	Utt-B+ Baf A1 12h	Utt-B 24h	Utt-B+ Baf A1 24h	Utt-B 48h	Utt-B+ Baf A1 48h
PARP	1.00	1.92	2.12	1.73	3.59	2.00	2.52	3.84
Cleaved PARP (p89)	1.00	4.78	6.20	6.12	8.19	5.72	10.73	1.64
Cleaved PARP (p24)	1.00	2.16	1.79	1.55	2.67	1.27	1.38	49.13

**Figure 3 K**	Control siRNA	Beclin siRNA
Beclin 1	1.00	0.16
LC3-II	1.00	0.27

Figure 4

**Figure 4 A**	C	5	10	15	20	25
LC3-II	1.00	1.29	1.79	1.69	1.67	1.55

**Figure 4 C**	C	Cqn	Utt-B	Utt-B+ Cqn
LC3-II	1.00	2.94	1.24	6.72

**Figure 4 H**	C	Cqn	Utt-B	Utt-B+ Cqn
Caspase 8	1.00	1.16	0.62	0.64
Cleaved casapase 8	1.00	0.85	0.37	1.07

**Figure 4 I**	C	Cqn	Utt-B	Utt-B+ Cqn
Caspase 9	1.00	0.45	0.72	0.77

Cleaved casapase 9	1.00	0.45	1.77	2.77
**Figure 4 J**	C	Cqn	Utt-B	Utt-B+ Cqn
PARP	1.00	1.34	1.36	1.39
Cleaved PARP (p89)	1.00	1.90	10.27	12.15
Cleaved PARP (p24)	1.00	2.28	7.99	9.30

Figure 6

**Figure 6 C**	C1	C2	C3	Cqn1	Cqn2	Cqn3	Utt-B1	Utt-B2	Utt-B3	Utt-B+ Cqn 1	Utt-B+ Cqn 2	Utt-B+ Cqn 3
Caspase 9	0.87	1.17	1.13	1.88	0.77	1.06	1.08	1.47	0.56	0.22	0.19	0.12
**Figure 6D**	C1	C2	C3	Cqn1	Cqn2	Cqn3	Utt-B1	Utt-B2	Utt-B3	Utt-B+ Cqn 1	Utt-B+ Cqn 2	Utt-B+ Cqn 3
PARP	1.18	0.47	0.20	0.38	0.35	0.15	0.40	0.30	1.64	1.09	1.19	1.03
Cleaved PARP (p24)	0.01	0.02	0.02	0.08	1.41	0.58	0.04	0.06	0.11	1.57	1.52	0.12

Supplementary Table 1. Quantification of western blots by ImageJ software
