# Supplementary material for: Blockade of Uttroside B-Induced Autophagic Pro-Survival Signals Augments Its Chemotherapeutic Efficacy Against Hepatocellular Carcinoma
Source: Front Oncol. 2022 Feb 8;12:812598. doi: 10.3389/fonc.2022.812598 (PMC8861526; doi:10.3389/fonc.2022.812598)
Supplement: Supplementary file 3 [file DataSheet_3.pdf]

Supplementary Table 2

Figure 2				
**Figure 2 J**	Control		Utt-B (10 mg/kg)	
p-mTOR (S2448)	104.90		2.09	
p-mTOR (S2481)	66.90		24.10	
**Figure2 K**	Control		Utt-B (10 mg/kg)	
p-Akt	42.20		38.76	
p-AMPKα	11.00		163.00	
**Figure2 L**	Control		Utt-B (10 mg/kg)	
p-4EBP-1	100.60		82.40	
p-p70S6 K	132.00		63.00	
**Figure 2 M**	Control		Utt-B (10 mg/kg)	
Beclin	116.00		151.00	
LC3-II	45.00		118.00	
**Figure 6**				
**Figure 6 F**	Control	Cqn (60mg/kg)	Utt-B (5mg/kg)	Utt-B+ Cqn
PCNA	75.5	26.6	154.9	232.7
Ki-67	23.4	50.8	88.1	130.9
**Figure 6 G**	Control	Cqn (60mg/kg)	Utt-B (5mg/kg)	Utt-B+ Cqn
LC3-II	56	71.3	69.2	90.5
Cleaved PARP	78.1	96.5	91.0	98.1

Supplementary Table 2. H-Score of IHC images analyzed using ImageJ software
